# Response to letter to the editor

**DOI:** 10.1002/alz.70251

**Published:** 2025-05-13

**Authors:** Kathy Y. Liu, Stephen Senn, Robert Howard

**Affiliations:** ^1^ Division of Psychiatry University College London London UK; ^2^ Sheffield Centre for Health and Related Research (SCHARR) The University of Sheffield Sheffield UK

1

Dear Editor,

We are pleased that Li and colleagues agree with our recommendation that “the average between‐group difference in score change is where the debate and research efforts should be focused to contextualize and evaluate the clinical meaningfulness of the true treatment effect.”

However, Li et al. do not address the central point of our article,^1^, ^2^ which was that the measurement of within‐individual variation attributable to treatment is precluded by the parallel‐arm randomized controlled trial (RCT) design typically employed in Alzheimer's disease (AD) treatment trials. This was why we argued that responder analyses risk committing “causal fraud” if differences in “responder” proportions (defined by a threshold of clinically meaningful change) are attributed to the treatment.

Instead, Li et al. implicitly endorse responder analyses of parallel‐arm trial data without clear statistical justification. Therefore, we would disagree with their statement that “statistically, MMRM is not inherently superior to responder analyses.” We would not support their inclusion of responder analyses among valid approaches to calculate a relative percentage reduction in outcome score decline to reflect the “underlying absolute treatment effect.”

Li et al. find it reassuring that various statistical approaches can result in relative percentage reductions in outcome score decline that “all converge on a similar magnitude.” In our view, this observation would not justify the use of any particular statistical approach, since the selection of appropriate statistical methods should be based on an ability to validly model the data. Furthermore, the serious pitfalls of using relative measures (such as relative percentage reduction, relative risk, hazard ratios) as the primary means of communicating RCT treatment outcomes, without considering the context of absolute differences, are well‐documented and widely recognized.^3^

Regarding the final statement in their Letter, in a parallel group RCT, time‐to‐event or progression‐free survival analyses can only validly provide group‐level comparisons and, therefore, cannot be used to infer individuals’ responses to treatment. For primary time‐to‐event or progression‐free survival outcome measures, the mean difference would still be *where the debate and research efforts should be focused to contextualize and evaluate the clinical meaningfulness of the true treatment effect*. Even where one has a case where a “change on the outcome can be accepted by most experts as clinically meaningful,” these experts would be deluding themselves if they thought they could identify which individuals benefited and which did not according to this standard.

## CONFLICT OF INTEREST STATEMENT

K.L. and R.H. declare no competing interests. S.S. acts as a consultant to the pharmaceutical industry but is unaware of any conflict of interest; a full list of his interests is maintained here: http://senns.uk/Declaration_Interest.htm. Author disclosures are available in the Supporting Information.

## Supporting information



Supporting Information
